# Rétinite à cytomégalovirus chez un patient atteint de rectocolite hémorragique sous azathioprine

**DOI:** 10.11604/pamj.2015.21.227.7489

**Published:** 2015-07-30

**Authors:** Wafa Ammari, Olfa Berriche

**Affiliations:** 1 Service d'Ophtalmologie, Hôpital Taher Sfar, Mahdia, Tunisie; 2 Service de Médecine Interne, Hôpital Taher Sfar, Mahdia Tunisie

**Keywords:** Rétinite, cytomégalovirus, rectocolite, Retinitis, cytomegalovirus, rectocolitis

## Image en medicine

Au cours des maladies inflammatoires chroniques de l'intestin (MICI), la fréquence de l'infection par le cytomégalovirus (CMV) est rare, variant entre 0,53% et 3,4%. Il s'agit le plus souvent d'une réactivation virale, plutôt que d'une primo-infection. La rétinite à CMV au cours d'une rectocolite hémorragique (RCH)est déjà rapportée, mais les liens de causalité ne sont pas clairement établis. Les capacités immuno-modulatrices du CMV et l'immunosuppression nécessaire au traitement de la maladie pourraient entretenir la réplication virale. Nous rapportons l'observation d'un patient âgé de 56 ans suivi pour RCH résistante, il était traité par azathioprine, quelques mois plus tard il se plaignait d'une asthénie avec une baisse de l'acuité visuelle, elle était évaluée de loin à 7/1O pour l’œil droit et 4/10 pour l’œil gauche. Le fond d’œil montrait la présence d'hémorragies et de nodules cotonneux à droite et un foyer de rétinite nasal actif, extensif à gauche. L'Angiographie à la fluorescéine objectivait une hypofluorescence rétinienne en faveur d'un foyer de rétinite profond gauche. Dans ce contexte d'immunodépression et devant l'aspect clinique évocateur, le diagnostic retenu était celui d'une rétinite à CMV dont le développement a probablement été favorisé par l'azathioprine. Le patient était mis sous Ganciclovir avec une évolution favorable.

**Figure 1:**
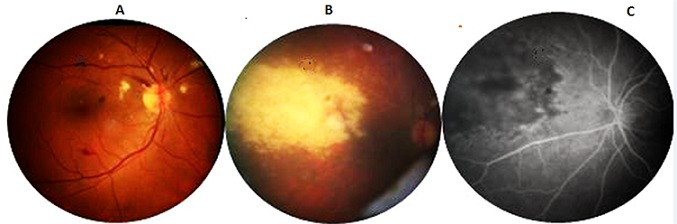
(A) fond d'oeil à droite: présence d'hémorragies et de nodules cotonneux; (B) fond d'oeil à gauche: foyer de rétinite nasal actif extensif; (C) angiographie a la fluorescéine: hypofluorescence rétinienne en faveur d'un foyer de rétinite profond de l'OG

